# Identification of QTLs controlling grain protein concentration using a high-density SNP and SSR linkage map in barley (*Hordeum vulgare* L.)

**DOI:** 10.1186/s12870-017-1067-6

**Published:** 2017-07-11

**Authors:** Chaofeng Fan, Huijie Zhai, Huifang Wang, Yafei Yue, Minghu Zhang, Jinghui Li, Shaozhe Wen, Ganggang Guo, Yawen Zeng, Zhongfu Ni, Mingshan You

**Affiliations:** 1 State Key Laboratory for Agrobiotechnology, Key Laboratory of Crop Heterosis and Utilization, Beijing Key Laboratory of Crop Genetic Improvement, China Agricultural University, Beijing, 100193 China; 2 National Plant Gene Research Centre, Beijing, 100193 China; 3 Institute of Crop Science, Chinese Academy of Agriculture Sciences, Beijing, 100081 China; 4 Biotechnology and Genetic Resources Institute, Yunnan Academy of Agricultural Sciences, Kunming, 650205 China

**Keywords:** Barley (*Hordeum vulgare* L.), Grain protein concentration (GPC), QTL, SNP marker, Near isogenic line

## Abstract

**Background:**

Grain protein concentration (GPC) is a major determinant of quality in barley (*Hordeum vulgare* L.). Breeding barley cultivars with high GPC has practical value for feed and food properties. The aim of the present study was to identify quantitative trait loci (QTLs) for GPC that could be detected under multiple environments.

**Results:**

A population of 190 recombinant inbred lines (RILs) deriving from a cross between Chinese landrace ZGMLEL with high GPC (> 20%) and Australian cultivar Schooner was used for linkage and QTL analyses. The genetic linkage map spanned 2353.48 cM in length with an average locus interval of 2.33 cM. GPC was evaluated under six environments for the RIL population and the two parental lines. In total, six environmentally stable QTLs for GPC were detected on chromosomes 2H (1), 4H (1), 6H (1), and 7H (3) and the increasing alleles were derived from ZGMLEL. Notably, the three QTLs on chromosome 7H (*QGpc.ZiSc-7H.1*, *QGpc.ZiSc-7H.2*, and *QGpc.ZiSc-7H.3*) that linked in coupling phase were firstly identified. Moreover, the genetic effects of stable QTLs on chromosomes 2H, 6H and 7H were validated using near isogenic lines (NILs).

**Conclusions:**

Collectively, the identified QTLs expanded our knowledge about the genetic basis of GPC in barley and could be selected to develop cultivars with high grain protein concentration.

**Electronic supplementary material:**

The online version of this article (doi:10.1186/s12870-017-1067-6) contains supplementary material, which is available to authorized users.

## Background

Protein is an essential nutrient for the survival of humans and animals [1, 2]. Protein in mature cereal grains, in particular, provides a substantial portion of the world’s plant protein, and its concentration determines the nutritional quality and end use properties of the grain [3, 4]. Barley (*Hordeum vulgare* L.) is one of the earliest domesticated crops in the world. Approximately 25% of its production has relatively lower GPC and is suitable for malting and brewing, while the remaining 75% with relatively higher GPC is used for feed and food (http://faostat.fao.org/). Hence, there is increasing need for breeding barley cultivars with high GPC, but this has been hindered by the relatively low heritability of GPC due to the significant interaction between environmental and genetic factors [5, 6]. Based on a statistical methodology, the genetic factors (quantitative trait loci, QTLs) that involved in determination of GPC can be elucidated [7]. Thus, identification and utilization of environmentally stable QTLs associated with GPC will provide an alternative but promising strategy for high GPC barley breeding.

To date, numerous studies have been conducted on dissecting the genetic basis of GPC, and QTLs have been mapped on all seven barley chromosomes. In particular, several consensus QTLs mapped on chromosomes 2H, 4H, 5H, 6H, and 7H have been repeatedly detected by multiple studies [8–15]. For example, two QTLs on chromosomes 5HS and 6HS located in the *Bmac0096*-*Bmag0323* and *ABG458*-*HVM74* intervals, respectively, have been repeatedly detected [10–12, 16]. Moreover, these two loci have also been identified by genome-wide association studies (GWAS) [17–19]. In addition, two genes (*HvNAM1* and *HvNAM2*) on chromosomes 6H and 2H in barley, which were suggested to be orthologous to *TtNAM-B1*, contributed a substantial effect on GPC [17, 20]. Notably, a recent study revealed that a single nucleotide polymorphism (SNP) within the second intron of *HvNAM2* was associated with GPC, which is useful in developing high quality barley cultivars [17]. Although these identified QTLs/genes for GPC that could be expressed under multiple environments might be valuable for GPC improvement in barley, most of the genetics studies focused on breeding and selection for low-protein barley [21, 22].

A saturated genetic linkage map will improve the precision of QTL localization and estimation of phenotypic variance, especially for some small and medium-sized QTLs [23]. Due to the abundance of SNPs in plant genome, SNP markers have been widely used in genetic linkage map construction [24–26]. High-density SNP linkage maps have been largely used in QTL detection for yield and quality in barley [27–29]. However, QTL mapping for GPC based on a high-density SNP map has rarely been reported. Here, to identify QTLs for GPC, a RIL population including 190 lines derived from a cross between the Chinese landrace ZGMLEL with high GPC (> 20%) and the Australian cultivar Schooner was used for linkage and QTL analyses. Furthermore, near-isogenic line (NIL) populations were developed to validate the environmentally stable QTLs.

## Methods

### Plant materials

A RIL population (generations F_9_ to F_11_) containing 190 RILs derived from two spring barley varieties, ZGMLEL and Schooner, was employed to identify QTLs controlling for GPC. ZGMLEL is a hull-less landrace with high GPC, while Schooner is a hulled cultivar with low GPC. All the RILs and their parental lines were kindly provided by Dr. Yawen Zeng (Yunnan Academy of Agricultural Sciences, China).

For NIL development, one RIL line (RIL7) was crossed with the recurrent parent (Schooner). Because the QTLs on chromosomes 6H (i.e. *QGpc.ZiSc-6H.2*, *QGpc.ZiSc-6H.3* and *QGpc.ZiSc-6H.4*) and 7H (i.e. *QGpc.ZiSc-7H.1*, *QGpc.ZiSc-7H.2* and *QGpc.ZiSc-7H.3*) were linked in coupling phase, the QTL clusters were introgressed into the Schooner background as a unity, respectively. After three backcross generations (BC_3_), individuals that solely exhibited heterozygosity at one QTL region were self-pollinated to produce its corresponding BC_3_F_2_ populations. Finally, three NIL populations, that is BC_3_F_2_-I (region 2H), BC_3_F_2_-II (region 6H) and BC_3_F_2_-III (region 7H), were developed for the validation their corresponding QTLs. The number of progenies in BC_3_F_2_-I, BC_3_F_2_-II and BC_3_F_2_-III populations were 249, 205 and 213, respectively.

### Field experiments

Field experiments were carried out in three locations, including Shangzhuang Experiment Station of CAU (China Agricultural University) in Beijing, Wangtaibao Experiment Station of NAAFS (Ningxia Academy of Agriculture and Forestry Sciences) in Ningxia Hui Autonomous Region, and Dishang Experiment Station of HAAFS (Hebei Academy of Agriculture and Forestry Science) in Hebei Province. The RIL population and the two parents were grown during three growing seasons from 2013 to 2015, providing data for six environments. Location-year information and corresponding weather data are presented in Additional file 1: Table S1. In field trials, each plot consisted of 2 rows that were 2 m long with approximately 20 plants per row. The middle ten plants in each line were bulk-harvested at maturity and measured for grain protein concentration (GPC).

Three BC_3_F_2_ populations for QTL validation were planted in Beijing (2016). Individuals were grown in 2-m-long rows with a 0.25-m row spacing. Within each row, 15 plants were evenly sown. At maturity, all the panicles were harvested from single-plant and sun-dried. Grain protein concentration (GPC), grain yield (GY) and thousand grain weight (TKW) were scored on a single-plant basis.

The field experiments were in accordance with local practice. All the trails were conducted under optimum irrigation. Nitrogen (N) was supplied at a rate of 220 kg/ha, including 70 kg/ha of N as diammonium phosphate and 80 kg/ha of N as urea applied before sowing. In addition, 70 kg/ha of N as urea was applied at booting stage.

### Phenotypic evaluation and statistical analysis

Mature grains of RIL population and BC_3_F_2_ populations were ground to a powder using a Cyclotec 1093 sample mill (Hoganas City, Sweden). Then, the ground powder was dried to a constant mass in an 80 °C oven. The total nitrogen content was determined using the Kjeldahl method with a FOSS Kjeltec ™ 2300 and then the GPC was calculated using a factor of 5.83 [30]. GY and TKW of the NIL populations were measured on a single-plant basis. TKW was determined using a camera-assisted phenotyping system, which was provided by Hangzhou Wanshen Detection Technology Co., Ltd. (Hangzhou, China).

The basic statistical analysis was performed using SPSS version 20.0 (SPSS, Chicago, IL, USA). The Shapiro-Wilk test was conducted using R software (V. 3.2.2) for the normality test. The best linear unbiased prediction (BLUP) for GPC across the six environments was calculated using SAS^®^ V.8 (SAS Institute Inc. 2000) with the PROC MIXED procedure. Under the random-effect model, environments were treated as fixed, and genotype and genotype-environments interactions were considered as random factors. The broad sense heritability (*h*_*B*_^2^) on a family basis was calculated using SAS^®^ V.8 (SAS Institute Inc. 2000) with the PROC GLM procedure, which was calculated according to the following formula: *h*_*B*_^2^ = *ó*_*g*_^2^ */ (ó*_*g*_^2^ *+ ó*_*ge*_^2^ */n + ó* ^2^ */nr)* where *ó*_*g*_^2^ = genotypic variance, *ó*_*ge*_^2^ = genotype by environmental variance, *ó* ^2^ = the residual error variance, *n* = the number of environments, and *r* = number of replicates.

### Linkage and QTL analyses

The RIL population was genotyped using the barley 9 K SNP chip developed from the RNA-seq data of barley varieties [31]. Additionally, a total of 21 polymorphic SSR markers were employed to genotype the RIL population, and most of the SSR sequences were obtained from http://wheat.pw.usda.gov/GG3/. Only markers with less than 5% missing data were selected for map construction. The genetic linkage map was constructed using RECORD 2.0 [32] and JoinMap 4.0 [33]. Markers with identical segregation were first removed using RECORD 2.0. After removing the redundancy, the unique markers were grouped using JoinMap 4.0 with a LOD value of 10. Finally, the marker order was established using the maximum likelihood mapping algorithm and the map distance was calculated using the Kosambi mapping function. The probe sequences of the SNP assigned to barley chromosomes were queried using the BLAST algorithm against barley reference genome sequence to locate chromosomal positions with a cutoff criterion of E-value ≤1e-10. The quality of the genetic map was validated using the alignments between SNP map and barley reference genome. Only the best hit of the SNP against the reference genome was selected for the collinearity analysis when the SNP was located to multiple paralogous positions in the genome.

The average GPC data in each environment and BLUP values across six environments were collected for QTL analysis. WinQTLCart2.5 software with the composite interval mapping (CIM) method was used to identify QTLs for GPC. The walking speed was set to 1 cM. Model 6 was chosen for QTL analysis, with 5 control markers and 10 cM window size defaults. The LOD threshold was set via 1000 permutations at *P* ≤ 0.05, and these QTLs were considered “identified QTLs”. A 2-LOD support with a 99% confidence level was chosen for each identified QTL. The identified QTLs detected in different environments with overlapping confidence intervals were regarded as the same in this study. The QTLs were named following the rules of Blake and Blake [34].

### Marker development

The flanking markers of the QTLs were employed to define the target region, which could be used to compare to the barley Genome Zipper developed by Mayer et al. [35]. Gene sequences of three grasses (rice, sorghum, and *Brachypodium*) were used as queries to blast against the database “assembly_WGSMorex” at IPK Barley BLAST Server (http://webblast.ipk-gatersleben.de/barley_ibsc/). The Morex contigs with best hit were employed to search for simple sequence repeat using the SSR Hunter software. Finally, the selected sequences were used to design SSR markers using the Primer3 software (http://frodo.wi.mit.edu/primer3/).

## Results

### Analysis of GPC

**Fig. 1:**
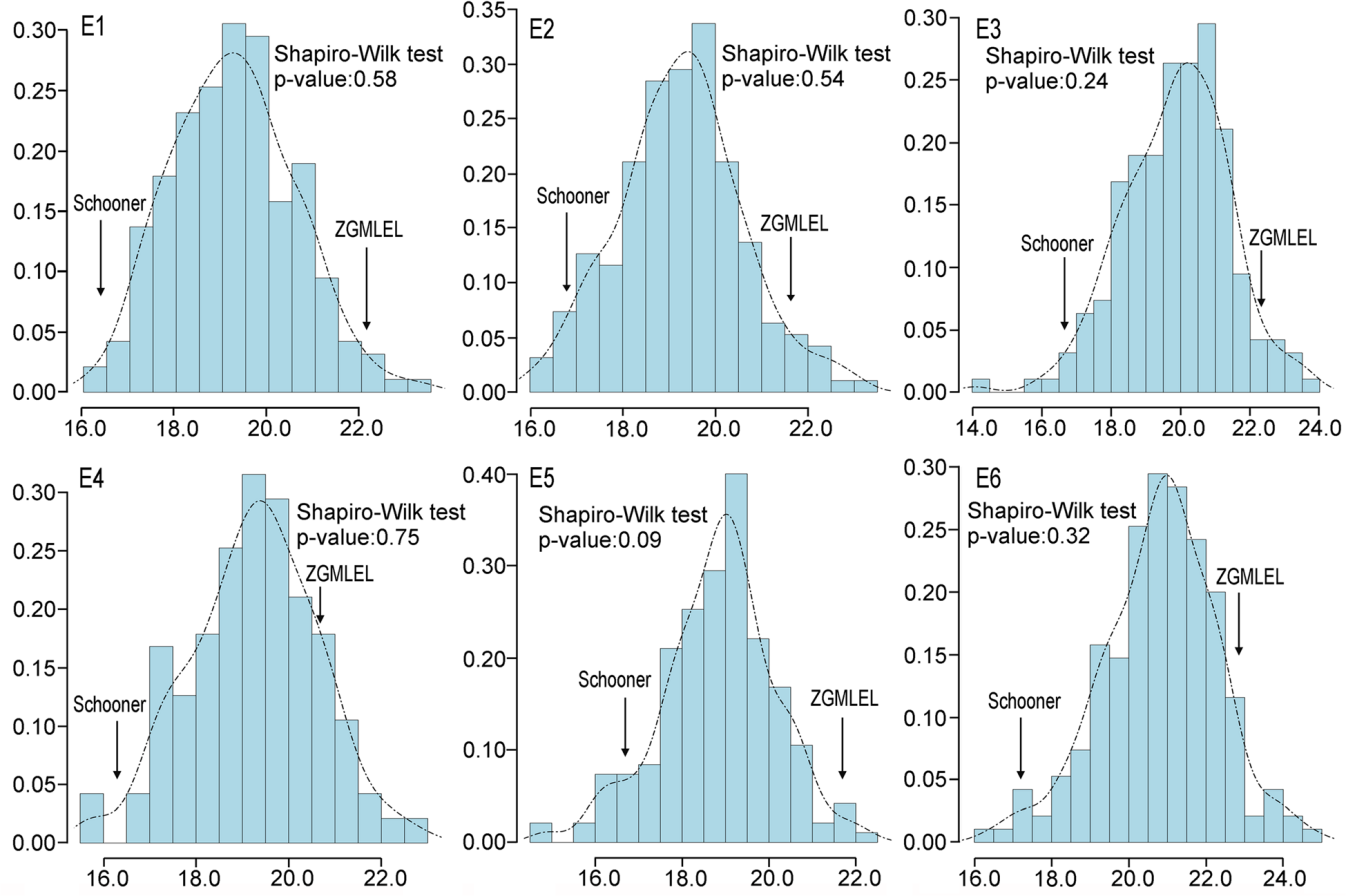
Histogram for grain protein concentration (GPC) in the ZGMLEL × Schooner population based on the mean value collected from six environments. The *Y*-axis represents the density of each group. The unit of the *X*-axis is GPC data

The basic statistics of minimum, maximum, mean standard deviation and coefficient of variation for GPC in six environments are listed in Table 1. The GPC of parent ZGMLEL ranged from 20.52% to 22.88% in the six environments evaluated, which was significantly higher (*P* < 0.01) than that of Schooner (16.35–17.20%) (Table 1; Additional file 2: Table S2). Moreover, the 190 RILs exhibited a wide range of variation in GPC, with coefficients of variation (CVs) ranging from 6.94% to 7.80% in the six environments. The Shapiro-Wilk for testing normality was performed for GPC based on the mean value collected from six environments (Fig. 1). In all of the six environments, GPC showed normal distribution, suggesting a quantitative nature of GPC in barley. Remarkably, the broad sense heritability (*h*_*B*_^2^) for the GPC of the RILs was 80.67%, indicating that the GPC variance was mostly determined by genetic factors.

**Table 1:** Parental and population minimums, maximums, means, standard deviations and coefficient of variations for grain protein concentration (GPC)

Environ. ^a^	Parental lines		RIL population					
	ZGMLEL	Schooner	Min.	Max.	Mean	SD ^b^	CV(%) ^c^	*h*_*B*_^*2*^(%) ^d^
E1	22.02 ± 0.53	16.45 ± 0.37	16.08	23.36	19.23	1.34	6.95	80.67
E2	21.54 ± 0.20	16.80 ± 0.62	16.01	23.05	19.25	1.36	7.07	
E3	22.41 ± 0.23	16.56 ± 0.19	14.08	23.75	19.87	1.55	7.80	
E4	20.52 ± 0.21	16.35 ± 0.25	15.72	22.93	19.25	1.38	7.18	
E5	21.70 ± 0.33	16.70 ± 0.26	14.78	22.28	18.83	1.31	6.94	
E6	22.88 ± 0.10	17.20 ± 0.09	16.35	24.83	20.80	1.50	7.22	

^a^E1, 2013-Beijing; E2, 2014-Beijing; E3, 2014-Hebei; E4, 2014-Ningxia; E5, 2015-Ningxia; E6, 2015-Hebei

^b^SD is the standard deviation

^c^CV is the coefficient of variation

^d^ *h*_*B*_^*2*^ is the broad sense heritability estimated across all six environments

### Construction of a high-density genetic linkage map

Of the 7864 SNP markers on the chip, 1526 (19.40%) were polymorphic between ZGMLEL and Schooner. After removing 53 SNPs with over 5% missing data, we used 1473 SNP markers and 21 polymorphic SSR markers to construct the linkage map. The resultant linkage map comprised nine linkage groups that contained 1011 unique loci and spanned 2353.48 cM. These linkage maps had an average locus interval of 2.33 cM (Table 2, Additional file 3: Table S3, Additional file 4: Figure S1). The identity and polarity of linkage groups were determined by BLAST against the barley reference sequence databases [36].

**Table 2:** Summary of the genetic linkage map constructed with the ZGMLEL × Schooner population

Chr.	No. of linkage groups	No. of markers	No. of loci	Length (cM)	Average locus interval (cM)	No. of loci assigned to barley genome	Covered physical length (Mb)	Total length of barley genome (Mb)	Coverage ratio (%)
1H	2	154/44	76/31	157.98/40.06	2.08/1.29	153/44	463.81 (0.25–464.06)	464.12	99.93
2H	1	302	203	468.20	2.31	291	411.63 (1.82–161.43, 372.38–624.40)	628.34	65.51
3H	1	203	112	204.37	1.82	196	472.31 (20.53–558.95)	564.43	95.40
4H	1	169	121	260.49	2.15	156	540.69 (0.03–540.72)	544.17	99.36
5H	2	125/65	97/51	314.67/146.14	3.24/2.87	116/57	354.43 (2.25–84.96, 285.64–557.36)	561.41	63.13
6H	1	252	184	322.40	1.75	237	342.26 (3.59–175.79, 363.74–533.80)	538.76	63.52
7H	1	180	136	421.17	3.10	161	599.46 (1.10–600.56)	601.60	99.64
Total	9	1494	1011	2353.48	2.33	1411	3184.59	3902.83	81.60

**Fig. 2:**
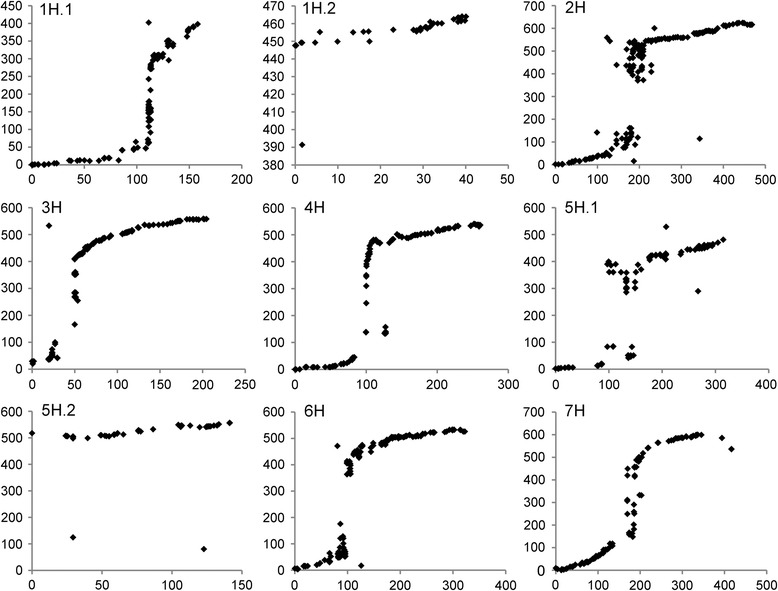
Alignments between the genetic linkage map in the ZGMLEL × Schooner population and barley physical map. The *X*-axis represents the genetic distance in centiMorgan (cM). The *Y*-axis represents the physical distance in million base pairs (Mb)

To further validate the quality of the map, SNP flanking sequences were employed to align with the barley reference sequence. Of 1473 SNP markers, 1411 (95.79%) were successfully assigned to the barley genome (Table 2; Additional file 5: Table S4). A good collinearity of the genetic map with the barley reference genome sequence was observed (Fig. 2), indicating a high quality of the genetic linkage map. However, several chromosome intervals were inconsistent with the reference genome sequence, i.e., chromosomes 2H at 76.43–204.46 cM, 4H at 125.91–127.33 cM, and 5H-1 at 100.55–146.27 cM.

Of the 5.1 Gb size of barley genome, 3.1 Gb has been successfully anchored to the physical map through population sequencing (POPSEQ) [36]. We calculated the coverage ratio for each barley chromosome. Chromosomes 1H, 3H, 4H and 7H displayed similar coverage ratios at 99.93, 95.40, 99.36, and 99.64%, respectively, and chromosomes 2H (65.51%), 5H (63.13%) and 6H (63.52%) exhibited lower ratios (Table 2).

### QTL mapping of GPC

**Fig. 3:**
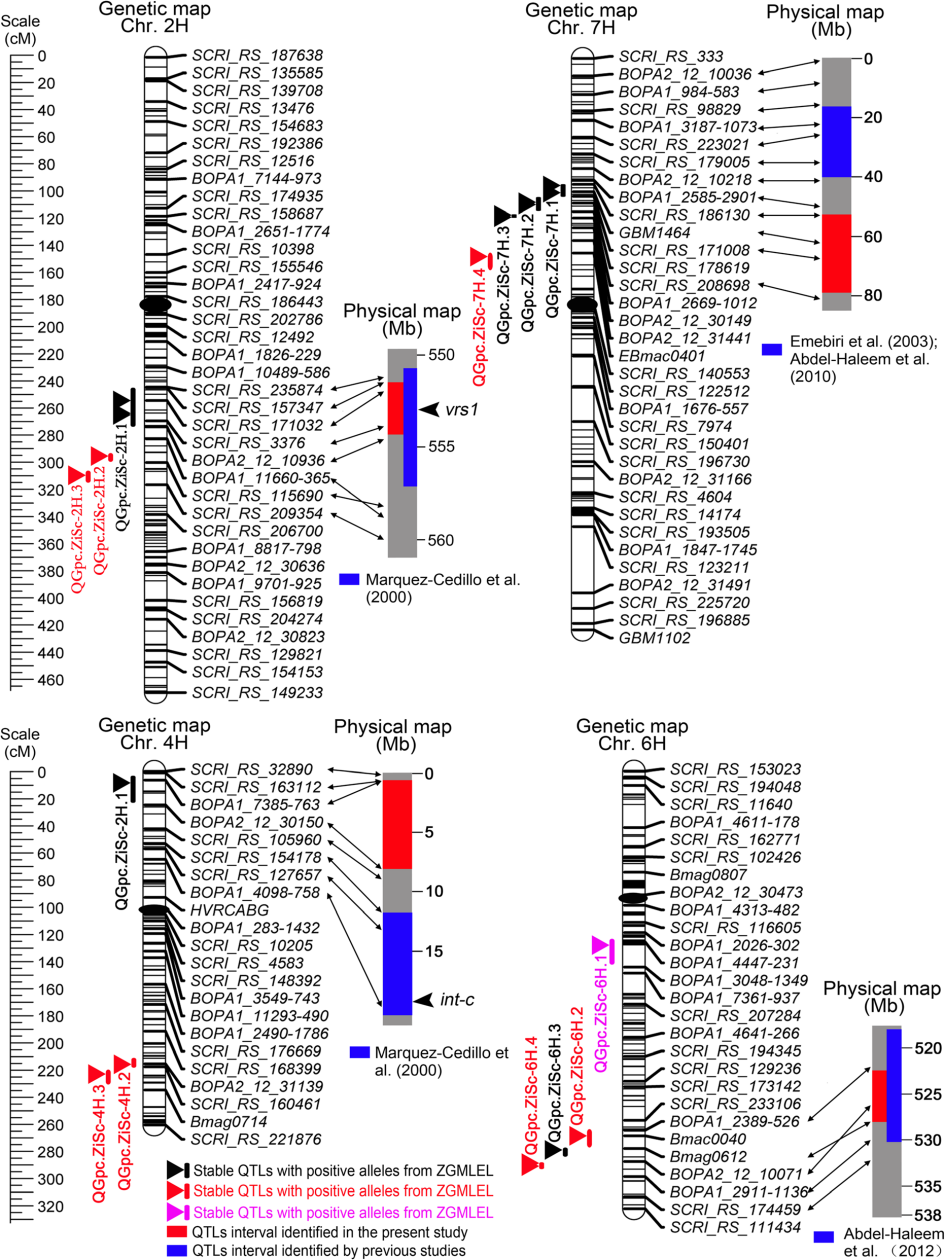
QTL locations for grain protein concentration (GPC) in the ZGMLEL × Schooner population. A centiMorgan (cM) scale is shown on the left. Vertical bar represents a 2-LOD interval for each QTL. Black ellipses represent the approximate locations of the centromeres. Black triangles indicate the environmentally stable with increasing allele from ZGMLEL. Red and pink triangles represent the putative QTLs that were detected only in less than three environments with increasing allele from ZGMLEL and Schooner, respectively. Blue and red shadows on the physical map represent the approximate positions of the QTL identified in previous studies and the present study, respectively. References from previous studies are presented under the physical map. The known positions of the *vrs1* and *int-c* loci are shown with black arrows (Ramsay et al. [72])

A total of 17 QTLs were detected for GPC, which are randomly distributed among chromosomes 2H (3 QTLs), 4H (3 QTLs), 5H (3 QTLs), 6H (4 QTLs), 7H (4 QTLs) (Fig. 3; Table 3). With an exception of *QGpc.ZgSc-6H.1*, ZGMLEL contributed effects for increased GPC at other 16 QTLs. These 16 significant QTL had LOD values ranging from 2.51 to 15.51 and explained the GPC variation from 2.4% to 19.86%. Schooner contributed effects for increased GPC at the locus of *QGpc.ZgSc-6H.1*, which had a LOD value of 3.37 and accounted for 5.70% of GPC variation. This indicated that the favorable alleles for increased GPC were mainly inherited from the feed barley ZGMLEL. In the present study, significant QTLs that could be detected in no less than three environments as well as in the combined analysis were defined as environmentally stable QTLs. According to this criterion, 6 of 17 significant QTLs were environmentally stable QTLs, which were identified on chromosomes 2HL (1), 4HS (1), 6HL (1), and 7HS (3).

**Table 3:** QTLs detected for grain protein concentration (GPC) in the ZGMLEL × Schooner population

QTL	Pos. (cM)	Nearest marker	LOD	*R* ^*2*^(%) ^a^	Additive ^b^	LOD2_interval	Environ.
*QGpc.ZiSc-2H.1*	253.8	*SCRI_RS_171032*	3.87	5.70	0.32	243.5–261.0	E1
	263.1	*SCRI_RS_126439*	3.28	3.70	0.26	252.2–270.4	E5
	263.1	*SCRI_RS_126439*	2.51	2.40	0.24	256.0–264.0	E6
	264.1	*SCRI_RS_126439*	3.78	4.47	0.23	259.9–271.7	C ^c^
*QGpc.ZiSc-2H.2*	294.3	*BOPA1_7236–1384*	3.77	5.69	0.34	291.2–298.7	E4
*QGpc.ZiSc-2H.3*	309.3	*SCRI_RS_170162*	5.14	8.58	0.41	305.3–314.4	E4
*QGpc.ZiSc-4H.1*	13.4	*BOPA1_7385–763*	3.65	5.80	0.38	2.5–23.9	E3
	14.4	*BOPA1_7385–763*	2.57	3.14	0.25	5.0–23.9	E4
	12.4	*BOPA1_7385–763*	3.33	5.26	0.35	2.6–14.9	E6
	12.4	*BOPA1_7385–763*	3.55	4.20	0.22	1.0–15.9	C
*QGpc.ZiSc-4H.2*	216.5	*BOPA2_12_31139*	4.58	5.68	0.33	212.3–217.6	E5
*QGpc.ZiSc-4H.3*	223.6	*SCRI_RS_160461*	5.59	6.10	0.34	219.1–228.6	E5
*QGpc.ZiSc-5H.1*	200.4	*BOPA1_9745–628*	4.71	7.08	0.41	188.6–206.7	E4
*QGpc.ZiSc-5H.2*	212.4	*BOPA1_3928–513*	4.13	6.56	0.39	206.7–217.6	E4
*QGpc.ZiSc-5H.3*	280.6	*BOPA1_10318–572*	4.12	4.86	0.30	272.9–285.9	E5
	282.6	*BOPA1_10318–572*	2.78	4.52	0.33	278.9–285.2	E6
	283.9	*SCRI_RS_218201*	3.31	3.37	0.20	278.9–285.2	C
*QGpc.ZiSc-6H.1*	146.4	*BOPA1_3048–1349*	3.37	5.70	−0.37	129.7–159.6	E2
*QGpc.ZiSc-6H.2*	275.7	*Bmac0040*	8.96	16.48	0.58	275.6–278.2	E2
	274.7	*Bmac0040*	10.09	16.44	0.58	269.7–278.2	E4
	275.7	*Bmac0040*	13.84	18.78	0.48	271.2–278.2	C
*QGpc.ZiSc-6H.3*	278.2	*Bmag0612*	9.16	13.42	0.51	278.0–283.3	E1
	280.2	*Bmag0612*	8.93	15.52	0.56	278.2–285.6	E2
	278.2	*Bmag0612*	4.67	7.01	0.43	278.2–283.9	E3
	282.2	*Bmag0612*	10.85	16.45	0.59	278.2–286.6	E4
	282.2	*Bmag0612*	15.51	19.86	0.61	277.6–286.6	E5
	278.2	*Bmag0612*	6.17	8.56	0.46	278.2–284.3	E6
	280.2	*Bmag0612*	13.82	17.90	0.46	278.2–283.8	C
*QGpc.ZiSc-6H.4*	290.7	*BOPA1_1852–509*	5.77	9.99	0.44	287.7–292.7	E1
	286.3	*SCRI_RS_124549*	5.64	8.45	0.46	287.3–292.3	E6
*QGpc.ZiSc-7H.1*	98.1	*GBM1464*	7.25	9.97	0.43	93.8–99.5	E1
	98.2	*GBM1464*	6.66	11.22	0.46	98.1–101.7	E2
	96.4	*SCRI_RS_152122*	3.19	4.44	0.30	95.0–100.2	E4
	98.2	*GBM1464*	5.83	6.38	0.34	98.1–100.2	E5
	96.4	*SCRI_RS_152122*	4.81	7.15	0.44	94.6–101.7	E6
	98.2	*GBM1464*	10.57	12.37	0.38	98.1–99.2	C
*QGpc.ZiSc-7H.2*	108.9	*SCRI_RS_138111*	9.47	15.43	0.56	107.1–111.9	E2
	106.8	*SCRI_RS_178619*	3.72	4.84	0.33	104.2–111.8	E4
	107.8	*SCRI_RS_178619*	6.58	7.51	0.38	104.7–111.5	E5
	109.9	*SCRI_RS_138111*	4.37	6.88	0.47	106.8–112.5	E6
	107.8	*SCRI_RS_178619*	10.71	13.29	0.41	107.0–112.2	C
*QGpc.ZiSc-7H.3*	116.0	*SCRI_RS_208698*	8.55	15.07	0.55	112.5–117.6	E2
	119.6	*BOPA1_2669–1012*	6.29	7.33	0.37	113.7–120.9	E5
	116.0	*SCRI_RS_208698*	3.35	5.67	0.42	113.0–117.0	E6
	116.0	*SCRI_RS_208698*	9.47	12.53	0.39	113.0–117.6	C
*QGpc.ZiSc-7H.4*	151.4	*EBmac0401*	5.11	7.48	0.47	143.8–155.6	E6

^a^ *R* ^*2*^ is the phenotypic variation explained by the identified QTL

^b^Positive value represents the increasing allele from ZGMLEL, while a negative value is from Schooner

^c^C is the combined QTL analysis based on the BLUP across six environments

Three QTLs associated with GPC were detected on chromosome 2HL. Only the one flanked by *SCRI_RS_171032* and *BOPA2_12_30901*, *QGpc.ZgSc-2H.1*, was considered being environmentally stable, which could be detected under three environments. *QGpc.ZgSc-2H.1* had a LOD value of 3.78 and explained 4.47% of the GPC variation for the combined analysis. The other two putative QTLs, *QGpc.ZgSc-2H.2* and *QGpc.ZgSc-2H.3*, were observed in one environment and explained 5.69–8.58% of GPC variation.

Among three significant QTLs for GPC detected on chromosome 4H, one stable QTL was identified on chromosome 4HS and designated *QGpc.ZgSc-4H.1*. This QTL had a minor effect, with a LOD value of 3.55, and explained 4.20% of the GPC variation for the combined analysis. *QGpc.ZgSc-4H.2* and *QGpc.ZgSc-4H.3* could be detected only in one environment, which had LOD and *R* ^*2*^ values ranging from 4.58 to 5.59 and 5.68% to 6.10%, respectively.

Chromosome 5HL carried three putative QTLs significantly associated with GPC, which were designated *QGpc.ZgSc-5H.3*, *QGpc.ZgSc-5H.1*, and *QGpc.ZgSc-5H.2*. *QGpc.ZgSc-5H.3* had a LOD value of 3.31 and explained 3.37% of the GPC variation for the combined analysis. The other two QTLs had the LOD values ranging from 4.13 to 4.71, and explained the GPC variation from 6.56% to 7.08%.

Four significant QTLs were identified on chromosome 6HL and were designated *QGpc.ZgSc-6H.1*, *QGpc.ZgSc-6H.2*, *QGpc.ZgSc-6H.3*, and *QGpc.ZgSc-6H.4*. Schooner contributed the effects for increasing GPC at *QGpc.ZgSc-6H.1*, and ZGMLEL contributed increased GPC at the other three loci. *QGpc.ZgSc-6H.3* was the most stable QTL for GPC, which could be detected in all six environments and explained as much as 17.90% of the GPC variation for the combined analysis. Two putative QTLs, *QGpc.ZgSc-6H.2* and *QGpc.ZgSc-6H.4*, were identified in two environments and explained 8.45–18.78% of GPC variation.

Four QTLs were found to be significantly associated with GPC on chromosome 7HS. Among these significant QTLs, three were environmentally stable QTLs, and they were designated *QGpc.ZgSc-7H.1*, *QGpc.ZgSc-7H.2*, and *QGpc.ZgSc-7H.3*. These three stable QTLs were detected in three to five environments, explaining 12.37–13.29% of GPC variation for the combined analysis. The last putative QTL, *QGpc.ZgSc-7H.4*, was detected at E6 and accounted for 7.48% of GPC variation.

### QTL validation

To study the three genomic regions on chromosomes 2H, 6H and 7H that possessing environmentally stable QTLs for GPC in more depth, three BC_3_F_2_ populations were developed and named BC_3_F_2_-I, BC_3_F_2_-II and BC_3_F_2_-III, respectively. Accordingly, three sets of SSR markers were used for foreground selection, i.e., *2L10*, *2L11* and *2L12* for BC_3_F_2_-I, *6L89*, *6L155* and *6L147* for BC_3_F_2_-II, and *7S40*, *7S69*, *7S87* and *7S89* for BC_3_F_2_-III (Additional file 6: Figure S2, Additional file 7: Table S5). A total of 76 SSRs were used for background selection of the BC_3_F_1_ individuals. Finally, three BC_3_F_1_ individuals that exhibit heterozygosity solely at genomic regions 2H, 6H or 7H were selfed to produce their corresponding BC_3_F_2_ populations. These three BC_3_F_1_ individuals shared 93.42, 92.10 and 94.74% similarities in genetic background with the recurrent parent, respectively.

To determine whether the stable QTLs affect the GPC in NIL populations, we compared the GPC between two homozygous groups. Based on the genotype of flanking markers (*2L10* and *2L11* on 2H, *6L89* and *6L147* on 6H, *7S87* and *7S40* on 7H), two homozygous groups, namely, ZGMLEL homozygous (ZZ) and Schooner homozygous (SS) were classified in each NIL population. The evaluation results for GPC showed that plants with ZZ genotype in BC_3_F_2_-I, BC_3_F_2_-II and BC_3_F_2_-III had an average GPC of 13.82, 14.18 and 14.20%, respectively. In contrast, plants with SS genotype in BC_3_F_2_-I, BC_3_F_2_-II and BC_3_F_2_-III had an average GPC of 13.15, 13.19 and 13.48%, respectively, which is similar to the recurrent parent, Schooner (13.32%) (Additional file 8: Table S6). Based on the GPC value, highly significant difference was found between two homozygous genotypes in each NIL population (*P* < 0.01) (Table 4, Additional file 8: Table S6). The allelic effects of the three populations were in the same direction as the original allele, with alleles from ZGMLEL increasing GPC. These results suggested that the stable QTLs on chromosomes 2H, 6H and 7H had significant effect on GPC, which was in agreement with the detection in RIL population.

**Table 4:** Variation between two homozygous genotypic groups of three NIL populations for grain protein concentration (GPC)

Population	GPC (mean ± SE ^c^) (%)		*P*-value	TKW (mean ± SE) (g)		*P*-value	GY (mean ± SE) (g)		*P*-value
	ZZ ^a^	SS ^b^		ZZ	SS		ZZ	SS	
BC_3_F_2_-I	13.82 ± 0.09	13.15 ± 0.05	2.40E-10	50.40 ± 0.19	50.55 ± 0.18	0.72	7.06 ± 0.12	6.74 ± 0.11	0.06
BC_3_F_2_-II	14.20 ± 0.11	13.47 ± 0.06	2.85E-08	50.44 ± 0.33	51.02 ± 0.25	0.22	6.79 ± 0.13	6.71 ± 0.10	0.58
BC_3_F_2_-III	14.18 ± 0.17	13.19 ± 0.12	5.33E-06	44.19 ± 0.40	45.82 ± 0.67	0.08	6.73 ± 0.23	6.40 ± 0.22	0.28

^a^ZZ represents ZGMLEL homozygote; ^b^ SS represents Schooner homozygote; ^c^ SE represents standard error

Previous studies reported that there was negative relationship between GPC and grain yield [37]. Thus, we measured thousand kernel weight (TKW) and grain yield per plant (GY) for the three BC_3_F_2_ populations but found no significant difference for TKW and GY (Table 4; Additional files 8: Table S6).

## Discussion

### The advantages and disadvantages of the present genetic linkage map

QTL mapping is a reliable way to resolve the genetic basis of GPC, and a high-density map will increase the accuracy of QTL detection [23]. In the present study, a high-density map comprised of 1473 SNP and 21 SSR, and spanned 2354.48 cM in length. Notably, our genetic map has a good collinearity with the barley reference genome, which is suitable for the identification of QTLs [38]. However, several chromosome intervals were inconsistent with the reference genome sequence. This could be partially explained by the following reasons: 1) a suppressed recombination frequency at the centromere region, 2) the presence of partially homologous sequences or duplication, and 3) the deficiency of polymorphic markers. In addition, four chromosomes (1H, 3H, 4H and 7H) had high genome coverage (95.40–99.93%), while three (2H, 5H and 6H) showed low genome coverage (63.13–65.51%), which might be caused by the lack of polymorphic markers within the centromeric region. Due to the low recombination frequency in the centromeric region, we speculated that it would not influence the identification of the QTLs.

Compared with two SNP maps reported by Close et al. [24] and Muñoz-Amatriaín et al. [25], the whole genome of our map expanded in genetic distance by 41.75 and 111.51%, respectively, with individual chromosome extended by 26.44% to 83.89% and 24.30% to 160.97%, respectively (Additional file 9: Table S7). Missing genotype data of each line and large number of heterozygotes could lead to expanded genetic distance [39, 40]. Consistent with this, similar phenomenon was observed in our SNP genotype data, which could partially contribute to the large whole genetic distance. The casual reason will be an interestingly area to further investigation.

### Extensive variation for GPC in barley

Determining the phenotypic variation of GPC in a segregating population is a prerequisite for elucidating its genetic foundation and for breeding barley cultivars with desirable GPC. Extensive variation in GPC in different barley genotypes has been reported previously. For example, analysis of 59 cultivated and 99 Tibetan wild barley accessions showed that the GPC ranged from 8.02% to 13.50% and Tibetan wild barley had much higher GPC than cultivated barley [17]. QTL analysis provides an efficient way to look for associations between the phenotypic variance and the markers segregating in a bi-parental population [41, 42] and has been widely used in dissecting GPC variation in barley populations. However, the lack of parental lines with high GPC in most previous studies may have hindered the detection of possible major QTLs for GPC [10, 43]. In the current study, the rare accession ZGMLEL, with consistently high GPC (20.52–22.88%), and an Australian cultivar, Schooner, with relatively low GPC (16.35–17.20%), were used to construct the mapping population. A relatively high broad sense heritability (80.67%) was found, suggesting that QTLs/genes controlling GPC are less environmentally influenced in the ZGMLEL × Schooner population. Thus, the ZGMLEL × Schooner population is a perfect material for identifying QTLs for GPC. Environmentally stable QTLs detected in this way might be suitable for marker-assisted selection (MAS) in barley breeding, which is anticipated to increase efficiency of the genetic improvement for GPC.

### Novel QTLs controlling GPC on chromosomes 4H and 7H

To enhance the GPC of barley, novel genes or QTLs with increased effects are of interest for breeding purposes. In our study, two genomic regions harboring four stable QTLs (*QGpc.ZgSc-4H.1*, *QGpc.ZgSc-7H.1*, *QGpc.ZgSc-7H.2*, and *QGpc.ZgSc-7H.3*) appeared to be novel (Fig. 3, Additional file 10: Table S8).

A significant QTL *QGpc.ZgSc-4H.1* for GPC was identified in the telomeric region of chromosome 4HS and was steadily expressed in three environments. QTLs affecting GPC have been identified on 4HS [8, 43, 44] and 4HL [15, 45, 46]. For example, Marquez-Cedillo et al. identified a QTL for GPC at the region of the *intermedium-c* (*int-c*) locus, which is obviously different from *QGpc.ZgSc-4H.1* (Fig. 3) [8]. Therefore, *QGpc.ZgSc-4H.1* likely represents a new locus for GPC, although its contribution to the variation of GPC was relatively small.

Remarkably, three neighboring QTLs (*QGpc.ZgSc-7H.1*, *QGpc.ZgSc-7H.2*, and *QGpc.ZgSc-7H.3*) were detected on chromosome 7HS. QTLs for GPC on chromosome 7H have been extensively reported [8, 12–14]. For example, Emebiri et al. [12] reported mapping of two QTLs for GPC in the telomeric and centromeric regions that are probably within the physical intervals of 15.8–40.0 and 261.8–277.6 Mb, respectively (Fig. 3) [12]. Marquez-Cedillo et al. [8] and Abdel-Haleem et al. [14] identified a consensus QTL near the *nud* locus on chromosome 7HL [8, 14]. However, the location of these QTLs was different from that detected in the present study. Therefore, these three QTLs in adjacent intervals are likely to be new QTLs, which might be due to the utilization of specific genetic materials in the present study.

### Consensus QTL regions for GPC on chromosomes 2H and 6H

An efficient method to introgress favorable alleles into elite germplasm is to select consensus QTLs that steadily affect GPC in different genetic backgrounds and environments [47]. For example, Emebiri reported that pyramiding two consensus QTLs on chromosomes 6HS and 5HS could significantly decrease GPC levels by 4% compared to the commercial check [22]. In this study, two genomic regions on chromosomes 2HL and 6HL for GPC were coincident with QTLs reported in previous studies (Fig. 3; Additional file 10: Table S8). For example, a stable QTL *QGpc.ZgSc-2H.1* on chromosome 2HL was coincident with the locus reported by Marquez-Cedillo et al. [8]. Another major QTL, *QGpc.ZgSc-6H.3*, explaining the highest GPC variance was mapped at a similar locus to *Qpro6a* detected in the Morex/Steptoe DH population [15]. However, the additive effect of our loci (0.43–0.59%), however, is higher than *Qpro6a* (0.14%), which might be caused by the special materials used in our study.

To date, two homologous genes, *HvNAM1* and *HvNAM2* associating with GPC on the short arm of chromosomes 6H and 2H, respectively, have been widely studied [17, 48, 49]. For example, Cai et al. [17] performed a multi-platform candidate gene-based association analysis using 59 cultivated and 99 Tibetan wild barley genotypes and found that the haplotypes of *HvNAM1* and *HvNAM2* markers were associated with GPC in barley. In the present study, two identified QTLs, *QGpc.ZgSc-6H.3* and *QGpc.ZgSc-2H.1* associated with GPC were also detected on chromosomes 6H and 2H, respectively, while they were both located on the long arms, demonstrating that *HvNAM1* and *HvNAM2* were obviously different from the QTLs detected in this study.

### QTLs for GPC linked in coupling phase on chromosomes 6H and 7H

Neighboring QTLs associated with many important traits, such as yield and quality, that are linked in coupling phase are commonly observed in primary QTL analysis [50, 51]. Previous studies have tried to dissect QTLs in coupling phase using nearly isogenic lines (NILs) or residual heterozygous lines (RHLs), and found that coupling QTLs were partially attributed to tightly linked independent QTLs [52–55]. For example, Han et al. identified two QTLs each for malt extract and for α-amylase and two to three for diastatic power in a complex QTL region using advanced segregation populations [53]. In this study, we detected two genomic regions on chromosomes 6HL and 7HS, each of which harbored linked QTLs for GPC. Region 6H contains three neighboring QTLs, i.e., one environmentally stable QTL (*QGpc.ZgSc-6H.3*) and two putative QTLs (*QGpc.ZgSc-6H.2* and *QGpc.ZgSc-6H.4*). These three QTLs with favorable alleles from one parent (ZGMLEL) were in coupling phase. A shadow QTL, significant but false, is caused by a real QTL in an adjacent marker interval [55]. Since *QGpc.ZgSc-6H.2* and *QGpc.ZgSc-6H.4* were located close to the stable QTL *QGpc.ZgSc-6H.3*, it is difficult to determine whether these two loci were shadow or genuine QTLs. Similarly, region 7H also contains three linked QTLs that were in coupling phase. Unlike the region 6H, region 7H harbored three environmentally stable QTLs and showed similar effects on GPC. Further studies are needed to dissect these two complex regions using advanced population.

### The contribution of stable QTLs to GPC

QTL effect was generally not precisely estimated in primary QTL analysis due to the genetic noise in mapping populations [56–62]. In view of this point, NILs were proposed and developed as an ideal population for QTL validation, especially for the QTL with a minor effect [63, 64]. In the present study, three genetic intervals harboring five stable QTLs, *QGpc.ZgSc-2H.1*, *QGpc.ZgSc-6H.3*, *QGpc.ZgSc-7H.1*, *QGpc.ZgSc-7H.2*, and *QGpc.ZgSc-7H.3* were identified and had additive effects of 0.23, 0.46, 0.38, 0.41, and 0.39% in the RIL population, respectively. Of these loci, three located on chromosome 7HS were linked together and resided in one genomic region. Three NIL populations, BC_3_F_2_-I, BC_3_F_2_-II and BC_3_F_2_-III, were developed according to a standard process of consecutive backcross, which targeted the genomic regions of chromosomes 2H, 6H and 7H, respectively. These stable QTLs were validated in the corresponding NIL population and their contribution to GPC could be directly compared between two homozygous groups in a similar genetic background. In populations I, II and III, the average GPC of plants carrying homozygous ZGMLEL were 0.66, 0.99 and 0.71% higher than that of plants carrying homozygous Schooner, respectively, providing further evidence for the reliability of these stable QTLs. Interestingly, the locus on chromosome 2HL (*QGpc.ZgSc-2H.1*) exhibited a strong potential increased in GPC in the Schooner background, which illustrated the conclusion that near-isogenic lines could be used to identify a quantitative locus even though it showed a relatively small effect on the phenotype [65]. Thus, the minor QTL, *QGpc.ZgSc-2H.1*, is feasible for cloning using NILs with least genetic noise. The estimation of the combination of three loci (*QGpc.ZgSc-7H.1*, *QGpc.ZgSc-7H.2*, and *QGpc.ZgSc-7H.3*) in BC_3_F_2_-III, however, was somewhat lower than expected by the sum of the individual effects of three loci, which might be caused by QTL × QTL interactions, QTL × environment interactions or QTL × new genetic background interactions.

A relatively lower GPC of BC_3_F_2_ populations (11.66–16.82%) grown in Beijing during year 2016 was observed as compared with the RIL population grown in Beijing during year 2013 (16.08–23.36%) or 2014 (14.08–23.75%). This provided further evidence that GPC is largely modified by environmental conditions, which may be due to the alteration of weather condition in 2016. Previous studies indicated high temperature during grain filling stage could result in enhanced GPC [66, 67]. Interestingly, the number of days after flowering with a maximum temperature above 30 °C in Beijing during 2013 and 2014 were 21 and 23 days, respectively, which is obviously more than that of 2016 (17 days) (Additional file 1: Table S1). Collectively, we speculated that the lower of GPC in 2016 may could be partially attributed to the alteration of temperature as compared to the other years.

### Potential application of stable QTL for MAS in barley breeding

Since grain protein concentration is greatly influenced by environmental factors, breeding high-GPC cultivars only through phenotypic evaluation has been proved to be less effective [68, 69]. Hence, selection of genomic regions containing QTLs that could express steadily under multiple environments is an efficient way to cultivate barley varieties [70]. Here, we report mapping of six environmentally stable QTLs for GPC that might be useful during barley breeding. Furthermore, we verified the effect of five stable QTLs located on chromosomes 2HL (*QGpc.ZgSc-2H.1*), 6HL (*QGpc.ZgSc-6H.3*) and 7HS (*QGpc.ZgSc-7H.1*, *QGpc.ZgSc-7H.2*, and *QGpc.ZgSc-7H.3*) using three NIL populations. In many cases, improvement of GPC is always accompanied by a significant reduction in grain yield [20, 71]. Notably, our preliminary data revealed that no significant difference was found for TKW and GY between two different homozygous groups for each QTL region. However, to further investigate the effect of QTL for GPC on yield, it is necessary to carry out experiment using lines, instead of single-plant strategy. Taken together, the identification of SSR marker intervals flanking these stable QTLs on chromosomes 2H (*2L10*-*2L12*), 6H (*6L89*-*6L147*) and 7H (*7S87*-*7S40*) may provide favorable regions for marker-assisted introgression into the elite barley germplasm.

## Conclusions

Based on genotyping 190 RILs in a genome-wide scale and measuring of GPC collected from six environments, six environmentally stable QTLs were significant associated with GPC, among which four QTLs on chromosomes 4H and 7H were firstly identified in the present study. Furthermore, three genomic regions harboring five stable QTLs on chromosomes 2H, 6H and 7H were validated using NIL populations, suggesting the reliability of QTLs detected in primary population. The markers linked to the stable QTLs would be valuable for MAS in barley breeding.

## Additional files

Additional file 1: Table S1. Location-year information and climate data for field trails. (DOC 39 kb)

Additional file 2: Table S2. Phenotypic data of two parental lines and 190 RILs under six individual environments and the combined analysis. (XLSX 31 kb)

Additional file 3: Table S3. Map locations of all markers and genotypic data of 190 RILs. (XLSX 1050 kb)

Additional file 4: Figure S1. Illustration of nine linkage groups constructed using 190 RILs. Notes: A centiMorgan (cM) scale is shown on the left. Black ellipses represent the approximate position for chromosome centromeres. The marker names are not shown. Detail information of the linkage group is provided in Additional file 3. (DOC 159 kb)

Additional file 5: Table S4. Alignments of SNP markers to barley reference sequence. (XLSX 76 kb)

Additional file 6: Figure S2. New genetic linkage maps in the target region of the stable QTLs detected in ZGMLEL × Schooner RIL population. (A) chromosome 2HL, (B) 6HL, and (C) 7HS. New SSR markers are showed in bold and underlined. (DOC 299 kb)

Additional file 7: Table S5. Markers developed to select the target regions of stable QTLs on chromosomes 2H, 6H and 7H. (DOC 38 kb)

Additional file 8: Table S6. Phenotypic data of two homozygous groups in each NIL population. (XLSX 36 kb)

Additional file 9: Table S7. Genome and chromosome size comparisons of this genetic map with previously reported maps. Notes: ^a^ the consensus map reported by Close et al. [24]; ^b^ the consensus map reported by Muñoz-Amatriaín et al. [25]. (DOC 38 kb)

Additional file 10: Table S8. Genomic regions harboring environmentally stable QTLs for grain protein concentration (GPC) in the ZGMLEL × Schooner population. Notes: QTLs in bold represent the environmentally stable QTLs. (DOC 35 kb)

## Abbreviations

GPC: Grain protein concentration
GY: Grain yield
MAS: Marker-assisted selection
NIL: Near-isogenic line
QTL: Quantitative trait locus
RIL: Recombinant inbred line
SNP: Single nucleotide polymorphism
SSR: Simple sequence repeat
TGW: Thousand grain weight
