# Supplementary material for: Identification of QTLs controlling grain protein concentration using a high-density SNP and SSR linkage map in barley (Hordeum vulgare L.)
Source: BMC Plant Biol. 2017 Jul 11;17:122. doi: 10.1186/s12870-017-1067-6 (PMC5504602; doi:10.1186/s12870-017-1067-6)
Supplement: Supplementary file 1 — Location-year information and climate data for field trails. (DOC 39 kb) [file 12870_2017_1067_MOESM1_ESM.doc]

**Table S1** Location-year information and climate data for field trails

| Location year | Code | Latitude,  longitude | Soil type | Planting date | Harvesting date | Average min temp (°C). | |  | Average max temp. (°C) | |  | Days max temp. >30 °C | |  | Precipitation (mm) | | Generation of the 190 RILs |
| --- | --- | --- | --- | --- | --- | --- | --- | --- | --- | --- | --- | --- | --- | --- | --- | --- | --- |
|  |  |  |  |  |  | pre-  anthesis | post-  anthesis |  | pre-  anthesis | post-  anthesis |  | pre-  anthesis | post-  anthesis |  | pre-  anthesis | post-  anthesis |  |
| Beijing, 2013 | E1 | 40°08' N, 116°10' E | Calcareous sandy loam | 2013/2/26 | 2013/6/25 | 4.05 | 18.07 |  | 15.85 | 29.87 |  | 0 | 21 |  | 16.2 | 109.0 | F_9_ |
| Beijing, 2014 | E2 |  |  | 2014/2/17 | 2013/6/20 | 6.19 | 17.28 |  | 17.58 | 29.49 |  | 0 | 23 |  | 22.9 | 112.6 | F_10_ |
| Beijing, 2016 |  |  |  | 2016/3/1 | 2016/4/30 | 6.52 | 17.02 |  | 18.89 | 28.76 |  | 0 | 17 |  | 55 | 370 | BC_3_F_2_ |
| Hebei, 2014 | E3 | 37°56' N, 114°42' E | Calcareous sandy loam | 2014/2/16 | 2014/6/17 | 7.52 | 19.40 |  | 17.53 | 30.34 |  | 0 | 29 |  | 28.9 | 37.4 | F_10_ |
| Hebei, 2015 | E6 |  |  | 2015/3/8 | 2015/6/17 | 9.13 | 18.99 |  | 20.70 | 30.19 |  | 5 | 27 |  | 47.0 | 40.7 | F_11_ |
| Ningxia, 2014 | E4 | 38°15' N, 106°15' E | Irrigation silted soil | 2014/2/27 | 2014/6/25 | 4.47 | 15.06 |  | 17.92 | 27.50 |  | 1 | 14 |  | 31.4 | 30.2 | F_10_ |
| Ningxia, 2015 | E5 |  |  | 2015/3/9 | 2014/7/1 | 5.60 | 15.68 |  | 17.17 | 27.17 |  | 2 | 14 |  | 29.9 | 7.0 | F_11_ |
