# Supplementary material for: Identification of QTLs controlling grain protein concentration using a high-density SNP and SSR linkage map in barley (Hordeum vulgare L.)
Source: BMC Plant Biol. 2017 Jul 11;17:122. doi: 10.1186/s12870-017-1067-6 (PMC5504602; doi:10.1186/s12870-017-1067-6)
Supplement: Supplementary file 4 — Illustration of nine linkage groups constructed using 190 RILs. Notes: A centiMorgan (cM) scale is shown on the left. Black ellipses represent the approximate position for chromosome centromeres. The marker names are not shown. Detail information of the linkage group is provided in Additional file 3. (DOC 159 kb) [file 12870_2017_1067_MOESM4_ESM.doc]

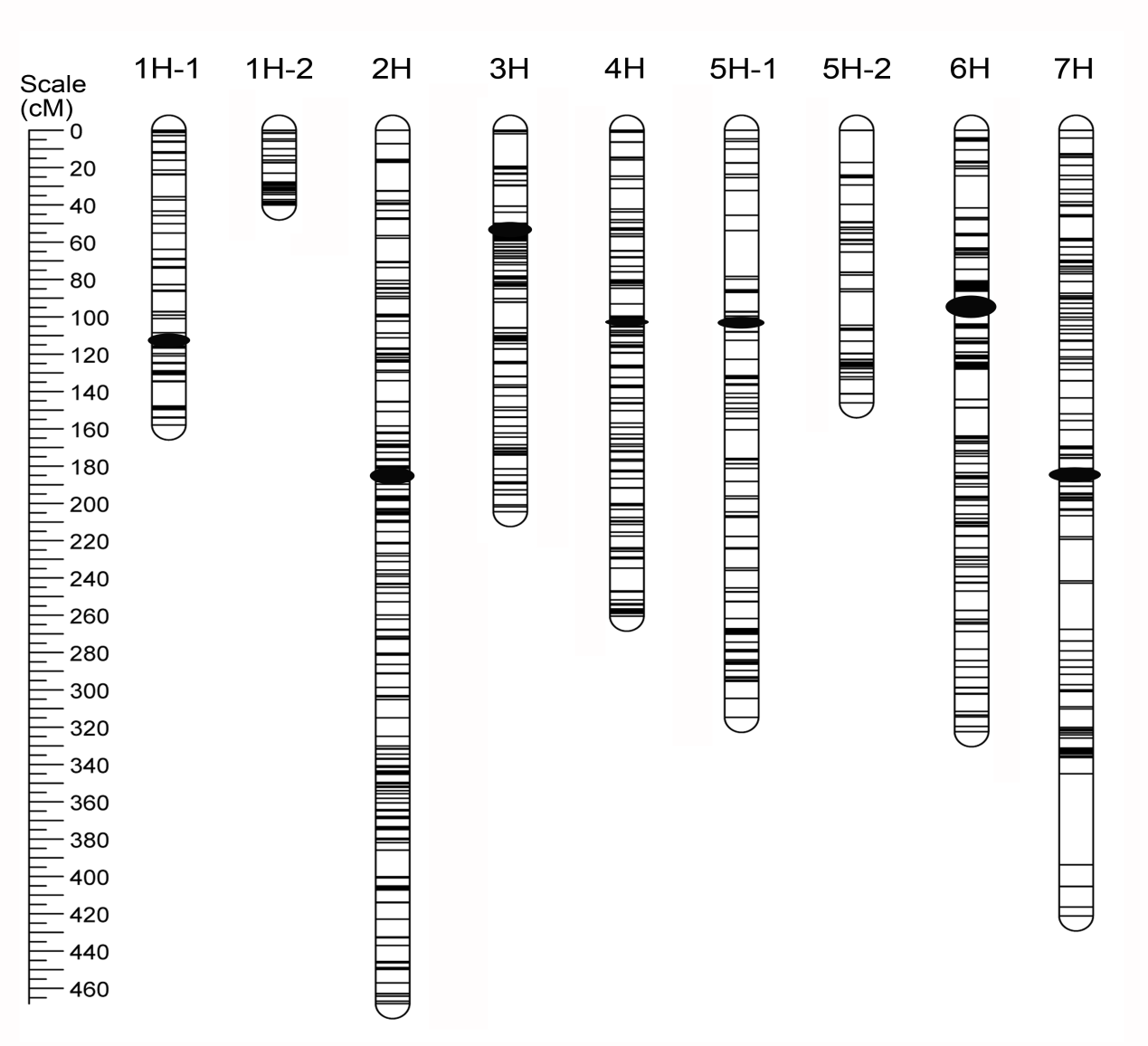


Figure S1 Illustration of nine linkage groups constructed using 190 RILs. A centiMorgan (cM) scale is shown on the left. Black ellipses represent the approximate position for chromosome centromeres. The marker names are not shown. Detail information of the linkage group is provided in Additional file 3.
