# Supplementary material for: Identification of QTLs controlling grain protein concentration using a high-density SNP and SSR linkage map in barley (Hordeum vulgare L.)
Source: BMC Plant Biol. 2017 Jul 11;17:122. doi: 10.1186/s12870-017-1067-6 (PMC5504602; doi:10.1186/s12870-017-1067-6)
Supplement: Supplementary file 6 — New genetic linkage maps in the target region of the stable QTLs detected in ZGMLEL × Schooner RIL population. (A) chromosome 2HL, (B) 6HL, and (C) 7HS. New SSR markers are showed in bold and underlined. (DOC 299 kb) [file 12870_2017_1067_MOESM6_ESM.doc]

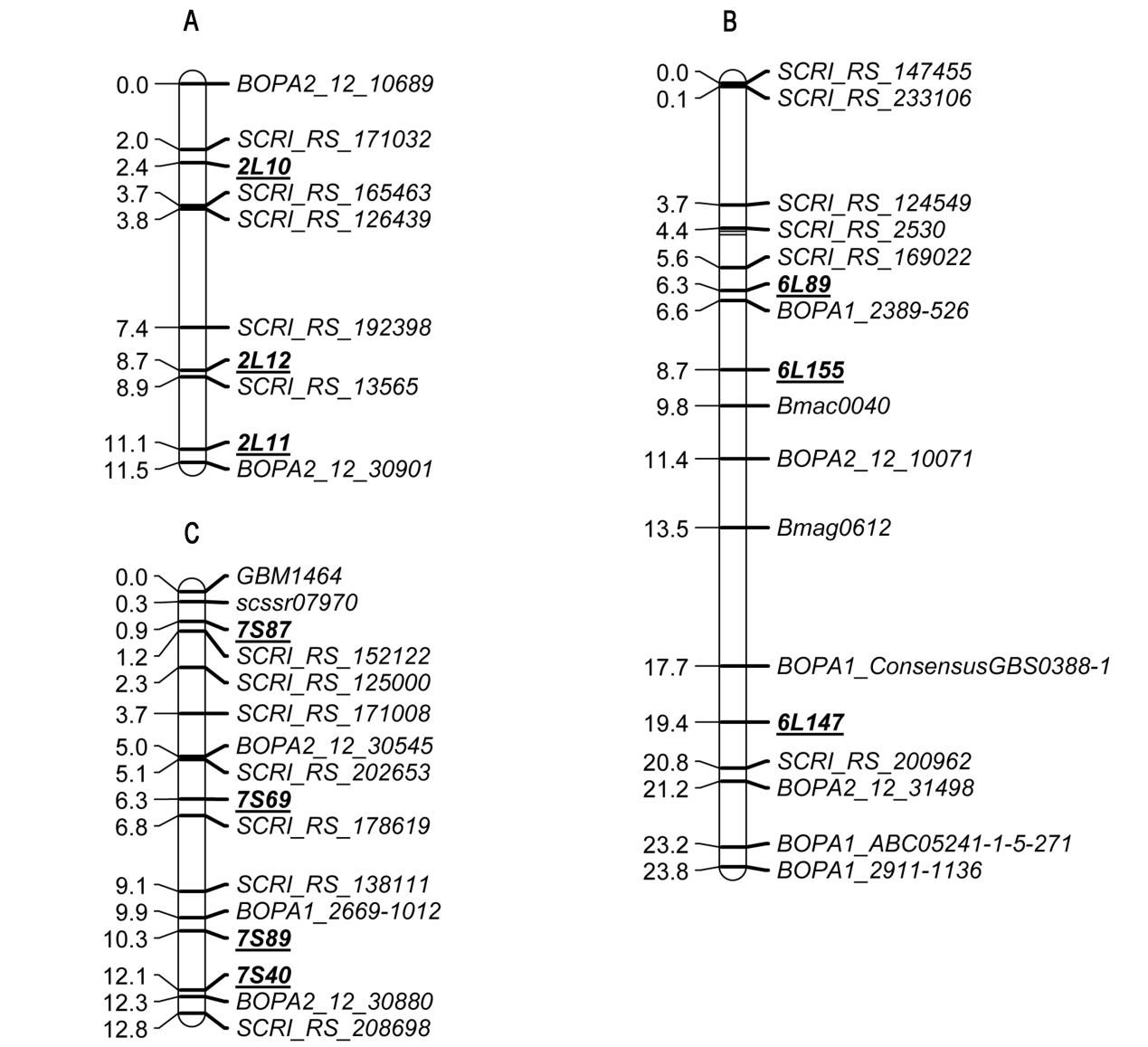


**Figure S2** New genetic linkage maps in the target region of the stable QTLs detected in ZGMLEL × Schooner RIL population. (A) chromosome 2HL, (B) 6HL, and (C) 7HS. New SSR markers are showed in bold and underlined.
