# Supplementary material for: Identification of QTLs controlling grain protein concentration using a high-density SNP and SSR linkage map in barley (Hordeum vulgare L.)
Source: BMC Plant Biol. 2017 Jul 11;17:122. doi: 10.1186/s12870-017-1067-6 (PMC5504602; doi:10.1186/s12870-017-1067-6)
Supplement: Supplementary file 7 — Markers developed to select the target regions of stable QTLs on chromosomes 2H, 6H and 7H. (DOC 38 kb) [file 12870_2017_1067_MOESM7_ESM.doc]

**Table S5 Markers developed to select the target regions of stable QTLs on chromosomes 2H, 6H and 7H**

| Name | Chr. | Forward primer | Reverse primer | Annealing temp. (°C) |
| --- | --- | --- | --- | --- |
| *2L10* | 2 | GCGCGGAAACTTTGCCTAAT | TGTCGTTTGCTTAGTGGGGA | 56 |
| *2L11* | 2 | CTGGTGAAGTATGTGCCCAA | TGGAGGGCTCAGTTACAAGG | 55 |
| *2L12* | 2 | ATCATCGTGCATTGTGACCC | TTCTATCACCGGGACTGAGC | 57 |
| *6L89* | 6 | AACTGTAGACACACCTGCCG | CAGGCAGCTGTTTCTTCCAT | 56 |
| *6L147* | 6 | AGCAAGTGCAGTCCAGTGAA | TTAGCCTTGCGGAGAGAGAG | 56 |
| *6L155* | 6 | CAGTCGAGAATGGGAATCGT | TGATGTCCGCACACAAAAAT | 56 |
| *7S40* | 7 | GGTGAGGAAGGAGGTGAGAG | TAGGGGTGAGTGTACGTTCG | 57 |
| *7S69* | 7 | CTTGCCATGTAAATGAGTTTT | GGGTCTTCCATTTCTACTGAG | 56 |
| *7S87* | 7 | TCCCAAATCTCTGCAGCATG | ACTTATAGTGCCATCCCGCT | 58 |
| *7S89* | 7 | AGGAACTGTCTGATTCTAGCC | GGTTTCCCTGTCCTAACTAAC | 56 |
