# Supplementary material for: Identification of QTLs controlling grain protein concentration using a high-density SNP and SSR linkage map in barley (Hordeum vulgare L.)
Source: BMC Plant Biol. 2017 Jul 11;17:122. doi: 10.1186/s12870-017-1067-6 (PMC5504602; doi:10.1186/s12870-017-1067-6)
Supplement: Supplementary file 9 — Genome and chromosome size comparisons of this genetic map with previously reported maps. Notes: a the consensus map reported by Close et al. [24]; b the consensus map reported by Muñoz-Amatriaín et al. [25]. (DOC 38 kb) [file 12870_2017_1067_MOESM9_ESM.doc]

**Table S7** Genome and chromosome size comparisons of this genetic map with previously reported maps

| Chr. | Genetic distance (cM) | | | Extension as compared the consensus map (2009) (%) | Extension as compared the consensus map (2014) (%) |
| --- | --- | --- | --- | --- | --- |
|  | Genetic map | 2009, consensus map ^a^ | 2014, consensus map ^b^ |  |  |
| 1H | 198.04 | 210.4 | 145.82 |  | 35.81 |
| 2H | 468.2 | 254.6 | 179.41 | 83.89 | 160.97 |
| 3H | 204.37 | 262.03 | 164.42 |  | 24.30 |
| 4H | 260.49 | 191.61 | 129.68 | 35.94 | 100.87 |
| 5H | 460.81 | 302.7 | 185.05 | 52.23 | 149.02 |
| 6H | 322.4 | 208.13 | 139.39 | 54.90 | 131.29 |
| 7H | 421.17 | 230.78 | 168.94 | 82.50 | 149.30 |
| Total | 2353.48 | 1660.25 | 1112.71 | 41.75 | 111.51 |

^a^ the consensus map reported by Close et al. (2009); ^b^ the consensus map reported by Muñoz-Amatriaín et al. (2014)
