# Supplementary material for: Identification of QTLs controlling grain protein concentration using a high-density SNP and SSR linkage map in barley (Hordeum vulgare L.)
Source: BMC Plant Biol. 2017 Jul 11;17:122. doi: 10.1186/s12870-017-1067-6 (PMC5504602; doi:10.1186/s12870-017-1067-6)
Supplement: Supplementary file 10 — Genomic regions harboring environmentally stable QTLs for grain protein concentration (GPC) in the ZGMLEL × Schooner population. Notes: QTLs in bold represent the environmentally stable QTLs. (DOC 35 kb) [file 12870_2017_1067_MOESM10_ESM.doc]

**Table S8** Genomic regions harboring environmentally stable QTLs for grain protein concentration (GPC) in the ZGMLEL × Schooner population

| Genomic region | QTLs ^a^ | Marker interval | Physical interval (Mb) | Marker interval of Ref QTL | Reference |
| --- | --- | --- | --- | --- | --- |
| Region 2H | ***QGpc.ZiSc-2H.1*** | *SCRI_RS_157347*-*BOPA2_12_30901* | 551.58-554.30 | *vrs1*-*MWG503* | Marquez-Cedillo et al. (2000) |
| Region 4H | ***QGpc.ZiSc-4H.1*** | *SCRI_RS_163112*-*BOPA2_12_30150* | 0.08-8.09 |  |  |
| Region 6H | *QGpc.ZiSc-6H.2* | *BOPA1_2389-526*-*BOPA1_1852-509* | 522.59-526.14 | *MWG934*-*MWG798A* | Abdel-Haleem et al. (2012) |
|  | ***QGpc.ZiSc-6H.3*** |  |  |  |  |
|  | *QGpc.ZiSc-6H.4* |  |  |  |  |
| Region 7H | ***QGpc.ZiSc-7H.1*** | *SCRI_RS_202061*-*BOPA1_2669-1012* | 52.97-91.82 |  |  |
|  | ***QGpc.ZiSc-7H.2*** |  |  |  |  |
|  | ***QGpc.ZiSc-7H.3*** |  |  |  |  |

^a^ QTLs in bold represent the environmentally stable QTLs
